# Heterogeneously-Integrated Optical Phase Shifters for Next-Generation Modulators and Switches on a Silicon Photonics Platform: A Review

**DOI:** 10.3390/mi12060625

**Published:** 2021-05-28

**Authors:** Younghyun Kim, Jae-Hoon Han, Daehwan Ahn, Sanghyeon Kim

**Affiliations:** 1 Department of Photonics and Nanoelectronics, BK21 FOUR ERICA-ACE Center, Hanyang University, Ansan 15588, Korea; younghyunkim@hanyang.ac.kr; 2 Center for Opto-Electronic Materials and Devices, Korea Institute of Science and Technology (KIST), Seoul 02792, Korea; daehwan23@kist.re.kr; 3 School of Electrical Engineering, Korea Advanced Institute of Science and Technology (KAIST), Daejeon 34141, Korea; shkim.ee@kaist.ac.kr

**Keywords:** Si optical phase shifter, optical modulator, optical switch, Si photonics, optical interconnect

## Abstract

The realization of a silicon optical phase shifter marked a cornerstone for the development of silicon photonics, and it is expected that optical interconnects based on the technology relax the explosive datacom growth in data centers. High-performance silicon optical modulators and switches, integrated into a chip, play a very important role in optical transceivers, encoding electrical signals onto the light at high speed and routing the optical signals, respectively. The development of the devices is continuously required to meet the ever-increasing data traffic at higher performance and lower cost. Therefore, heterogeneous integration is one of the highly promising approaches, expected to enable high modulation efficiency, low loss, low power consumption, small device footprint, etc. Therefore, we review heterogeneously integrated optical modulators and switches for the next-generation silicon photonic platform.

## 1. Introduction

Silicon (Si) photonics is a mainstream technology of optical interconnection, mainly for an optical transceiver, to meet the ever-increasing data traffic, which has greatly increased due to the coronavirus [1]. This technology has been dramatically researched and developed in the past several years. It enables the large-scale integration of variable optical functionalities on a chip with the advantage of cost-effectiveness, thanks to the use of existing Si CMOS process facilities and its technology [2,3,4,5,6]. As mentioned above, demand for optical transceivers in data centers has been driving the development of Si photonics to mature. On the other hand, other applications for optical sensing and computing have emerged which take advantage of this technology, e.g., phased array for Lidar system [7,8,9,10] and matrix multiplication unit for artificial intelligence [11,12,13,14,15].

One of the key components in the applications is an optical phase shifter, which controls optical signals. It is highly important because it is a fundamental function on large-scale Si photonic integrated circuits (PICs). Optical phase shifters have been developed for more than a decade with the development of Si optical modulators because they are essential cells of the modulators. For a conventional Si optical modulator, the free-carrier effect is commonly used to modulate the refractive index of Si because Si has no significant electro-optic effect [16,17]. In the early stage, carrier-injection type modulators have been largely developed thanks to the large free-carrier effect and relatively easy fabrication process, but it suffers from limited device speed due to minority carrier lifetime. Although there have been reports on carrier-injection type high-speed modulators based on a pin junction, an additional function, e.g., pre-emphasis signals, for driver circuits, is required to supplement its limited electro-optical response [18]. The most common operation mechanisms for high-speed modulation are carrier depletion using a p-n junction [19,20,21,22,23,24] and carrier accumulation using a metal-oxide-semiconductor (MOS) capacitor [25,26,27], which are embedded in a Si waveguide, composing an interferometer or a resonator. In particular, the free-carrier effect-based Si Mach-Zehnder modulators (MZMs) at a data rate of 200 Gb/s and 400 Gb/s IEEE Ethernet standard, taking advantage of 4-level pulse amplitude modulation (PAM-4) [28] and quadrature phase-shift keying (QPSK) [29] have been demonstrated.

However, the weak plasma dispersion effect in Si causes low modulation efficiency. *V_π_L*, the product of the voltage and the phase-shifter length for a π-phase shift, is the important parameter for phase shifters, indicating the modulation efficiency (therefore, small *V_π_L* implies high modulation efficiency). The typical *V_π_L* for a carrier-depletion Si MZ modulator is 1.5–2.5 Vcm, making the phase shifter length greater than 1 mm. Therefore, the low modulation efficiency results in a device length of around several millimeters for MZ modulators [3,29,30,31,32,33]. Although the modulation efficiency can be reduced by increasing free-carrier densities of the p-n junction, it results in high optical loss due to free carrier absorption. Hence, it is important to improve the modulation efficiency taking into account the tradeoff relationship between the modulation efficiency and optical phase-shifter loss [34]. On top of that, there have been many efforts to improve modulation efficiency. Compared to conventional Si MZ modulators, Si micro-ring modulators using a high-cavity resonator have been proposed which greatly reduce the footprint to <10 µm diameter [35,36,37,38]. Slow light-assisted Si modulators using a photonic crystal with more than 10 times smaller size have been demonstrated [39,40]. Surface plasmon-polariton-based modulators have been attracted taking advantage of the strong optical confinement [41]. However, they usually suffer from high insertion loss. To mitigate these limitations, many researchers have devoted lots of efforts in very recent years to material engineering. Among them, heterogeneous integration on Si is one of the most promising solutions. Hybrid Si optical phase shifters, SiGe, III-V, LiNbO_3_, and BaTiO_3_ on Si, have been investigated to enhance the modulation efficiency.

Optical switches are also one of the important active components on a Si photonics platform. To achieve switching operation on large-scale Si PICs, an electro-optic plasma dispersion effect [42], a thermo-optic effect [43], or an actuated micro-electro-mechanical systems (MEMS) [44] are commonly used. Although a plasma dispersion effect using an optical phase shifter is also commonly used for high-speed optical switching, it has relatively high crosstalk compared to other switches [42,43,44,45,46,47,48]. A thermo-optic effect using a metal heater and an actuated mechanical structure using MEMS can achieve relatively low crosstalk; however, they suffer from low speed, relatively high-power consumption, or high driving voltage [42,43,44,45,46,47,48]. In the case of an optical switch using an electro-optic optical phase shifter, its characteristics and trade-off relationship are mostly the same with the optical modulator. Additionally, other structures have their trade-off relationship; thus, we will also discuss the main trade-off relationships of these structures.

In this paper, we review heterogeneously integrated Si optical phase shifters. The paper is organized as follows: Section 2 reviews hybrid Si optical modulators and switches. Section 3 discusses both devices and benchmarks all different types of modulators and switches presented in Section 2. Finally, Section 4 concludes the paper.

## 2. Optical Phase Shifters

Optical phase shifters have the functionality to control the phase of light by a change in the effective refractive index. In a Si photonics platform, it is an essential part composing optical modulators as well as switches in Si photonics. Here, we review those devices in which the heterogeneous integration contributes to enhancing device performance.

### 2.1. Optical Modulators

Datacenter connections are the main driver for the R&D of Si photonics technology due to the ever-increasing data rate. They are moving from 25/100G to 100G/400G and optical transceiver modules have been developing toward 400G and beyond. In terms of that, Si optical modulators play an important role to satisfy the data rate. The development of modulators has been making it possible to take advantage of advanced modulation formats [28,29]. However, it is necessary to leverage heterogeneous integration for further improvement due to the limitation of Si’s poor electro-optic effect.

The properties of the light in Si waveguides are changed through a change of the optical constants of the waveguides, refractive index, and absorption coefficient. There are mainly two methods to change the effective refractive index, which are the thermo-optic effect and the plasma dispersion effect. The former is usually used in optical switches which don’t require the GHz regime high-speed operation and the latter is mostly used for high-speed Si modulators.

The plasma dispersion effect and free-carrier absorption are expressed by the Drude model, describing that changes in refractive index and absorption coefficient arise from a change in the plasma frequency of free carriers which is dependent on the number of free carriers and their conductivity effective masses. Thus, the changes in refractive index (Δ*n*) and absorption coefficient (Δ*α*) are expressed by

Δ*n* = −(*e*^2^*λ*^2^/8*π*^2^*c*^2^*ε*_0_*n*)[Δ*N_e_*/m*_ce_ + Δ*N_h_*/m*_ch_] (1)

Δ*α* = (*e*^3^*λ*^2^/4*π*^2^*c*^3^*ε*_0_*n*)[ΔN_e_/m*_ce_^2^*μ_e_* + ΔN_h_/m*_ce_^2^*μ_h_*] (2)

where *e* is the electronic charge, *ε*_0_ is the permittivity in a vacuum, *c* is the speed of light in vacuum, *λ* is the wavelength, *n* is the unperturbed refractive index, m*_ce_ and m*_ch_ are the conductivity effective masses for electron and hole, and *μ_e_* and *μ_h_* is the mobilities for electron and hole, respectively [16].

As shown in Equations (1) and (2), the parameters are physical constants except for the change in free-carrier densities. For Si, Soref and Bennett evaluated the changes in optical constants based on experimentally investigated results [16] as shown in Equations (3) and (4) for 1.55-μm wavelength.

Δ*n* = −[8.8 × 10^–22^ × Δ*N_e_* + 8.5 × 10^−18^ × (Δ*N_h_*)^0.8^] (3)

Δ*α* = 8.5 × 10^–18^ × Δ*N_e_* + 6.0 × 10^−18^ × Δ*N_h_* (4)

**Figure 1:**
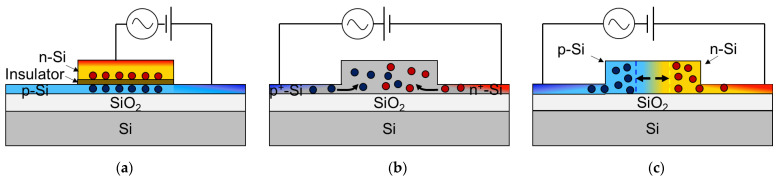
Cross-sections of typical device structures implementing the three different mechanisms (**a**) Carrier accumulation, (**b**) Carrier injection, and (**c**) Carrier depletion.

There are three different types of Si optical modulators as shown in Figure 1. Electrical manipulation of free carriers interacting with the propagating light in the Si waveguide is achievable based on the types such as carrier accumulation, carrier injection, and carrier depletion. Carrier-accumulation and carrier-depletion types consist of semiconductor-insulator-semiconductor (SIS) and *pn* junction, respectively. They are more suitable for the high-speed modulation due to majority-carrier-based operation, compared to carrier-injection type comprised of *pin* junction. While the carrier accumulation type modulator requires an oxide barrier, the carrier-depletion type only requires a highly CMOS-compatible *pn* junction. Therefore, the latter is the most common approach due to relatively easy fabrication and simple design.

The *V_π_L* of carrier-depletion type modulators with a horizontal *pn* junciton is as high as 1.5 to 2.5 Vcm [3,29,30,31,32,33,34]. Although the modulation efficiency can be reduced further by increasing free-carrier densities of the *pn* junction, it results in high optical loss due to free-carrier absorption [34]. In terms of that, the different shapes of vertical, L-shape, and U-shape *pn* junctions can provide the larger modulation efficiency maintaining optical loss due to the larger mode overlap between the optical mode and larger junction area of the *pn* junctions [49,50], but the larger junction area increases the junction capacitance, limiting modulation speed. The comparative analysis on optical modulators of both Si lateral and L-shape junctions has been reported. The tradeoff between static performance, *V_π_L,* and phase-shifter loss, and dynamic performance, *f*_mod_, is comparatively and quantitatively well investigated. In addition, such L-shape *pn* junction-based modulators should be carefully designed considering *f*_mod_, sacrificing *V_π_L* to achieve target dynamic optical modulation amplitude (OMA) [34].

**Figure 2:**
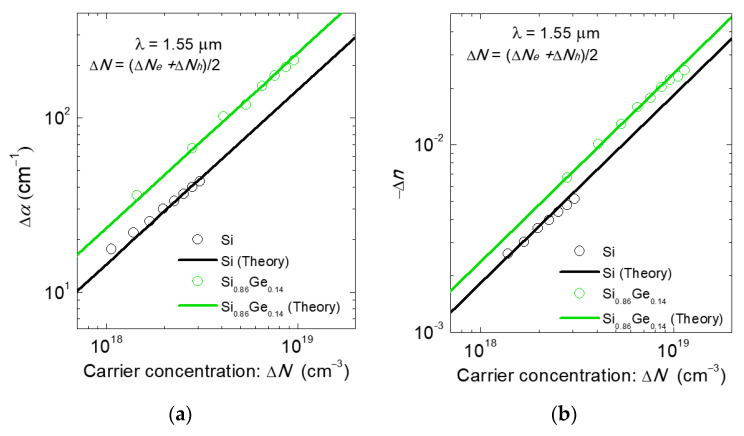
Changes in optical constants of Si_0.86_Ge_0.14_ and Si as functions of carrier concentration. (**a**) Change in refractive index and (**b**) Change in absorption coefficient [53].

As can be seen in Si modulators above, it is important to introduce heterogeneous integration, taking advantage of materials with superior electro-optic effects in conjunction with an Si photonics platform. One of the materials is silicon germanium (SiGe). As shown in the plasma dispersion effect, Equation (1), the change in refractive index is inversely proportional to the effective masses of electrons and holes. Therefore, the lighter the conductivity masses become, the greater the plasma dispersion is. In CMOS technology, the application of strain to Si has been widely employed to achieve lighter conductivity masses and higher mobilities in the channels of transistors, which can improve MOS transistor’s performance. Tensile strain and compressive strain are introduced to Si for n-channel and p-channel MOS transistors, respectively. It was also experimentally reported that the plasma dispersion effect and free-carrier absorption in Si in the far-infrared wavelength range from 5 to 20 μm were modified by uniaxial strain mechanically applied to Si through strain-induced mass modulation [51,52]. On the other hand, strained SiGe is well-known as a p-channel material in CMOS technology due to its high hole mobility originated from the low effective mass of holes [53,54,55,56]. Therefore, it is expected to enhance the plasma dispersion effect. Y. Kim et al. experimentally showed strain-induced enhancement of plasma dispersion effect in silicon-germanium (SiGe) optical modulators. Figure 2 presents that the plasma dispersion effect and free-carrier absorption are enhanced by strain-induced mass modulation in strained SiGe.

**Figure 3:**
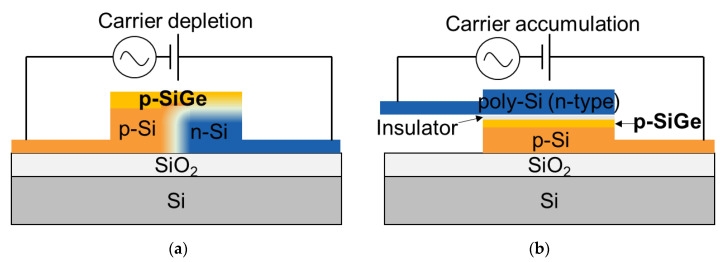
(**a**) Schematic cross-sections of (**a**) carrier depletion type [57] and (**b**) accumulation type [58] modulators using a strained p-SiGe layer to enhance plasma dispersion effect.

In 2018, Fujikata et al. demonstrated high-speed and highly efficient Si carrier-depletion type MZ modulators with a thin and strained SiGe layer [57]. The device cross-sections are shown in Figure 3a. The change in carrier profiles for carrier depletion in p-SiGe and n-Si regions is induced by applying reverse bias voltages e.g., under bias conditions of 0 and −2 V. p-SiGe is selectively grown on a Si waveguide and the crystal defects are barely shown in the Transmission Electron Micrograph (TEM) image in the reference. Based on the device performances of *V_π_L* (0.67 Vcm, −0.5 V), 3-dB bandwidth (12 GHz at −1 V), and insertion loss (1.5 dB to 2.0 dB), the high-speed operation of 25 Gbps was achieved at near 1.3-μm wavelength. The *V_π_L* is lower than typical lateral *pn* junction Si modulators. Since the optical mode is near the center of the waveguide, it would be better to redesign the SiGe position so that mode overlap in SiGe can increase.

Another method of heterogeneous integration is to apply the SIS structure for accumulation-based capacitive modulators. Additionally, it is more suitable for low driving voltage operation and power consumption thanks to the lower *V_π_L*, compared to typical Si *pn* junction-based modulators, which require traveling wave or multi-stage electrodes, and represent an extra-energy cost. M. Takenaka et al. proposed SiGe capacitive optical modulators as well as the theoretical background strain engineering of the enhanced plasma dispersion effect [54]. In addition, it was demonstrated by M. Douix et al. in 2019, shown in Figure 3b [58]. Since strained SiGe technology is already used in CMOS technology, it was fabricated on a 300-mm Si photonics platform. As shown in Figure 3b, SiGe is selectively grown on top of the bottom patterned Si-on-insulator (SOI) waveguide, followed by insulator deposition. Then, the top side waveguide is made up of poly-Si deposition. The position of the insulator was designed to be the center of the waveguide where the optical intensity is maximum, indicating optical mode overlap in SiGe is nearly maximized. The Si capacitive modulator without an SiGe layer was also prepared to compare with the device with SiGe. The phase shift against waveguide width and applied DC bias, with and without the strained SiGe layer was used for the comparison. SiGe device presents a 20% enhancement in efficiency due to higher hole efficiency in strained SiGe, resulting in *V_π_L* as low as 0.27 Vcm with 4-nm oxide thickness. However, they showed dynamic characteristics of the SiGe device which *V_π_L* is 1.08 Vcm with 13-nm taking into account an optimal tradeoff between device length, optical modulation amplitude, and electrical RC constant. The modulator response is limited to ~4 GHz, mainly limited by an access resistance. If optimized, device response is expected to be 10 times faster than the demonstrated device. In 2021, I. Charlet et al. have recently reported the optimized SiGe device can achieve 0.59 Vcm for *V_π_L* and 37 GHz for 3-dB bandwidth, resulting in the dynamic OMA of −3 dBm at scattering propagation loss, 8 dB/cm [59].

**Figure 4:**
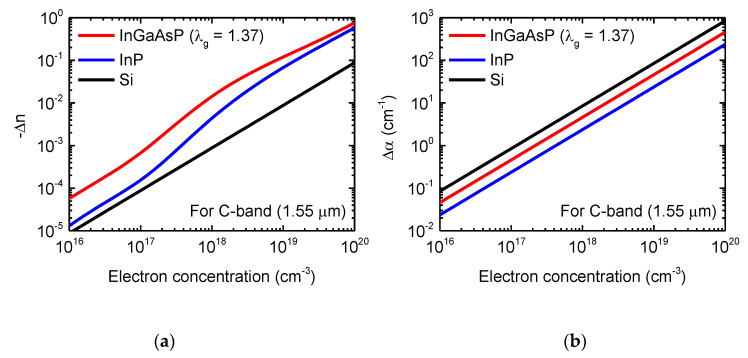
Numerical analysis of an InGaAsP/Si hybrid MOS optical modulator (**a**) Electron-induced refractive index changes (Δ*n*) of InGaAsP, InP and Si, (**b**) Electron-induced absorption changes (Δα) of InGaAsP, InP and Si [61].

One main drawback of SiGe is that free-carrier absorption is also increased by strain-induced effective mass modulation with an increase in the plasma dispersion effect, which was also experimentally reported and shown in Figure 2b. In terms of that, III-V materials have the advantageous inherent property in which free-carrier absorption is reduced while the plasma dispersion effect is enhanced for electrons [60,61,62,63,64]. In 1990, B. R. Bennett et al. investigated the carrier-induced change in the refractive index of III-V compound semiconductors, InP, GaAs, and InGaAsP [60]. Since the plasma dispersion effect is inversely proportional to the effective mass as shown in Equation (1), the light electron effective mass in III-V materials results in a greater change in the refractive index than that in Si. In addition, the bandfilling electro-optic effect and bandgap shrinkage contribute to the changes in the refractive index and absorption of n-type III-V materials. When the free carriers increase, the plasma dispersion effect and the bandfilling effect contribute to a negative refractive index change, while the bandgap shrinkage contributes to a positive change. Therefore, the plasma dispersion effect and the bandfilling effect are dominant for phase modulation. Importantly, they predicted the low-loss optical phase modulators and switches based on carrier modulation. In 2017, J. Han et al. demonstrated a highly efficient and low-loss InGaAsP/Si hybrid SIS-based capacitive modulator [61]. They also reported the theoretical model, in which the change in refractive index is larger for InGaAsP and InP than Si, while the change in absorption coefficient is smaller than Si, as shown in Figure 4a,b, respectively.

**Figure 5:**
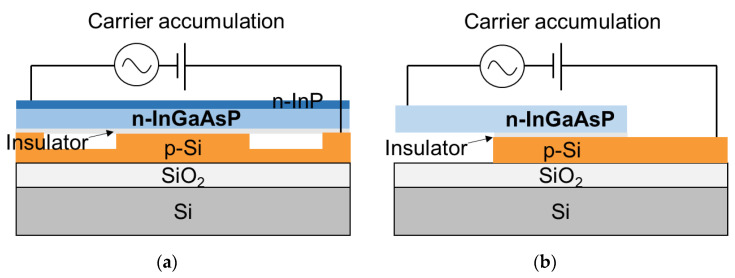
(**a**) Cross-section of an InGaAsP/Si hybrid MOS optical phase shifter demonstrated by J. Han et al. [61], and (**b**) of InGaAsP/Si MOS capacitor phase-shifter demonstrated by T. Hiraki et al. [62].

To heterogeneously integrate III-V on the Si rib waveguide, they used the wafer bonding technique. An InP substrate containing InGaAsP/InP layers was bonded on the Si waveguide using an Al_2_O_3_ bonding interface. Figure 5a shows the device cross-section of the modulator, indicating optical mode is positioned near the SIS interfaces. They also investigated and optimized the interface quality to achieve a steep capacitance-voltage curve, which highly affects optical modulation. With 5-nm equivalent oxide thickness (EOT), they achieved a very low *V_π_L* of 0.047 Vcm due to InGaAsP and thin EOT. The phase shifter loss is around 20 dB/cm. Thanks to the low *V_π_L*, the device exhibited an extremely small attenuation of 0.23 dB for the 500-μm-long phase shifter. It is noticeable that the *V_π_L* and attenuation levels are one-order magnitude better than the conventional Si modulators. The experimental dynamic characteristics were not presented due to their high resistance and capacitance because the device was not optimized for high-speed operation. However, based on simulations, they mentioned that a high-speed modulation is also possible for the optimized III-V MOS modulator by showing eye diagrams of a 53-Gbaud 4-level pulse-amplitude modulation (PAM-4).

In the same year of 2017, T. Hiraki et al. also demonstrated heterogeneously integrated InGaAsP MOS capacitor MZ modulators [62]. Figure 5b shows the device cross-section the device was fabricated by the wafer bonding technique as well. Compared to J. Han et al.’s demonstration, it is more practically designed, taking into account the tradeoff between SiO_2_ thickness and RC delay so that it can operate GHz-regime modulation. Therefore, the *V_π_L* of 0.09 Vcm, is higher, but the cutoff frequency of ~2.2 GHz is faster than that in J. Han’s work. This dynamic performance enables a 32 Gbps modulation with signal pre-emphasis. They recently reported a high-efficiency MZ modulator integrated with a DFB laser using membrane InP-based devices on an Si photonics platform. The latest work [63] shows a higher *V_π_L* of 0.4 Vcm compared to their previous work, but the measured 3-dB bandwidth is around 31 GHz which is much more practical for high-speed modulators. Finally, they demonstrated eye diagrams with a 50-Gbps NRZ signal using the integrated DFB laser diode and high-efficiency InGaAsP MZM. From this work, it is important to design SIS-type modulator parameters, e.g., capacitance of an insulator, considering target data rate and bandwidth rather than demonstrating just extremely low *V_π_L*. It is feasible for switch applications but not high-speed modulators.

The introduced heterogeneous III-V SIS modulators above are fabricated through wafer bonding with thin bonding layers and require metal contacts to the III-V material to realize the desired III-V-insulator-Si capacitive phase-shifter structures. Such process steps are quite challenging and less CMOS compatible, which may result in lower manufacturability and scalability. Integration of III-V on the Si platform through epitaxy has been investigated for the next-generation electronic and photonic devices in a CMOS pilot line [65,66]. Y. Kim et al. proposed the carrier-depletion monolithic III-V/Si optical phase modulator, leveraging the direct growth of III-V on Si V-groove [67], and S. Kim reported its performance as predicted by TCAD simulations [68]. The simulation study suggests the feasibility of a very low *V_π_L* of 0.07 Vcm, a low phase-shifter loss of 16 dB/cm, and a very low α*V_π_L* product close to 1VdB at 1.31 μm, which is 10 times lower than for typical Si *pn* optical phase shifters. One of key issues is a defect-induced optical loss for a low loss phase shifter because it is difficult to achieve low enough defect density at the III-V/Si interface, where an optical mode exists.

**Figure 6:**
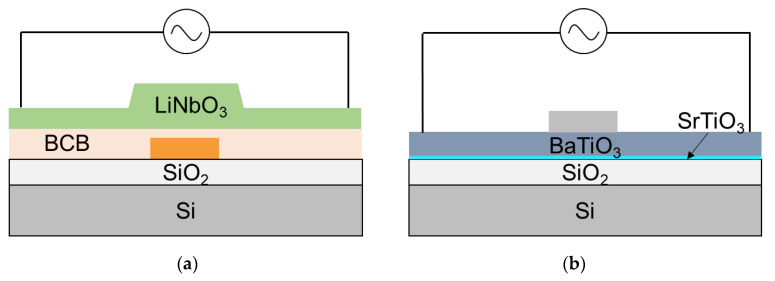
(**a**) Schematic cross-sections of (**a**) LN/Si optical phase shifter [73] and (**b**) BTO/Si optical phase shifter using Pockels effect [75].

Unlike the free-carrier effect, an applied electric field can change a material’s refractive index and so modulate the light using a linear electro-optic phenomenon known as the Pockels effect. The Pockels effect has none of the constraints of the free-carrier effect, which are unavoidable carrier induced loss due to free-carrier absorption and limited operating speed due to carrier lifetime for carrier injection type or RC delay for carrier depletion type. However, Si has almost no Pockels effect since it does not exist in a centrosymmetric crystal, indicating that it is difficult for Si to take advantage of the Pockels effect. Therefore, the leveraging of such materials with a non-centrosymmetric crystal structure and strong Pockels effect on a Si photonics platform for high-performance optical phase shifters has been investigated. Lithium niobate, LiNbO_3_ (LN), with a Pockels coefficient around ~30 pm/V, is one of the most popular materials for electro-optical modulators with the role of external modulation in fiber-optic transmission systems. The LN modulators are typically fabricated using LN substrates and titanium diffusion and proton exchange are used for LN waveguide formation with a refractive index contrast of ~0.04, which is much smaller than that in Si photonics. Therefore, the off-the-shelf LN modulators are bulky and power-consuming with a large *V_π_L* of more than 10 Vcm. There have been many efforts to make bulky LN modulators integrated so that they can support capacity-hungry short reach links such as data-center interconnects. For high refractive index contrast and flexibility in fabricating nanostructures, a thin-film lithium niobate platform was proposed [69], and high-performance coherent optical modulators were demonstrated on the LN-on-insulator platform [70,71,72]. Furthermore, the hybrid approach of Si and LN MZ modulators was demonstrated by M. He et al. in 2019 [73]. Figure 6a shows the schematic of the hybrid Si/LN modulator, which was fabricated by the die-to-wafer using benzocyclobuten (BCB) adhesive and LN dry etching technique. The modulator is composed of passive components of Si grating couplers, Si MMI couplers, and Si waveguides, and active components of LN-based phase-shifters. One of the important parts, which enable it to be hybrid, are the vertical adiabatic couplers (VACs), which transfer the optical power between Si and LN membrane waveguides (>97%, loss of ~0.19 dB per VAC). The hybrid Si/LN modulator with the LN phase shifter length of 5 mm exhibits an insertion loss of 2.5 dB, *V_π_L* of 2.2 Vcm, electro-optic bandwidth of at least 70 GHz, and modulation rates up to 112 Gbps.

Simply, if the stronger Pockels effect can be used, a smaller device footprint is available. Regarding the strong Pockels’ material, barium titanate, BaTiO_3_ (BTO) crystals have a Pockels coefficient of 1600 pm/V, more than 50 times higher than LN. In addition, they can also be epitaxially grown as thin films on an Si substrate. Therefore, BTO emerges as a highly promising material to achieve Pockels electro-optic modulators integrated on a Si photonics platform [74,75,76,77]. First, S. Abel et al. demonstrated a large Pockels effect in micro- and nanostructured BTO integrated on Si [74], as shown in Figure 5b. They fabricated photonic and plasmonic devices employing BTO growth on SOI wafers by molecular beam epitaxy and wafer bonding and verified the Pockels effect to be the physical origin of the response, with the Pockels coefficient tensor element, *r*_42_ = 923 pm/V. F. Eltes et al. reported a monolithically integrated BTO-based optical modulator on a Si photonics platform targeting a next-generation platform [75]. The same method reported in [74] was used to integrate BTO into EPIC. The only difference is the host wafer which is an electronic and photonic integrated circuits (EPIC) processed substrate, instead of a bare SiO_2_/Si wafer. The integrated BTO/Si MZ modulator shows excellent *V_π_L* of 0.2 Vcm and α*V_π_L* of 1.3 VdB (~0.7 VdB when optimized), indicating ~5.7 dB/cm (~3.0 dB/cm when optimized) and works at high speed of 25 Gbps, which is expected to reach data rates >50 Gbps by an adapted electrode design. Here, one feature of the optical modulators based on the Pockels effect are that they can be operated at cryogenic temperatures, whereas free-carrier effects-based optical modulators are restricted at low temperatures [76]. This is expected to encourage the use of Si photonics for quantum computing in the future.

### 2.2. Optical Switches

Optical switches are also important components for large-scale data center interconnections [45]. Although free-space optical switches realized by micro-electromechanical systems (MEMS) [44,46] and liquid crystal on silicon (LCOS) [47] have been already commercialized for large-scale data center application, these approaches have disadvantages, such as high cost owing to its high process complexity and the difficulty of high-volume manufacturing [45,48,78,79,80]. To overcome these problems, lithography-based fabrication, especially CMOS-compatible integrated photonics, has been widely investigated [45,48,78,79,80].

**Figure 7:**
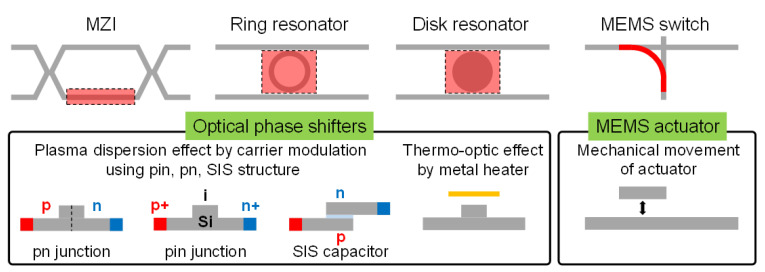
Schematics of various optical switches in large-scale Si PICs.

To achieve integrated optical switches, several approaches have been suggested. Although electro-optic effects are the main modulation principles for the optical modulators [42], other principles, such as a thermo-optic effect [43] or MEMS actuators [44], are also widely used for optical switches to achieve high scalability and low crosstalk. In the case of the electro-optic optical and thermo-optic switches, an optical phase shifter is implanted into the interferometer or resonator structures as shown in Figure 7; thus, efficient modulation schemes discussed in previous captures are also partially effective for high-performance optical switches. On the other hand, the MEMS actuators of MEMS optical switches made by poly-Si or silicon-on-insulator (SOI) are mechanically moved to connect each optical pass. In this section, the recent progress on the integrated optical switches is discussed.

The thermo-optic switches [81,82,83,84] and MEMS optical switches [85,86,87,88] are commonly investigated to achieve large scalability and low crosstalk. These structures have relatively small losses compared to the electro-optic effect due to no carrier response; thus, they can achieve small crosstalk. Particularly in the case of MEMS optical switches, it is easier to design a large-scale switch array with small complexity and crosstalk because they need no multi-stage interferometers or resonators [89]. However, the optical loss caused by structure parameters, such as the distance between a metal heater and the waveguide [90] and the distance between a MEMS actuator and the waveguide [91,92], is the main drawback of these structures. Relatively low speed and large power consumption are also the main issues on these devices. MEMS optical switches can also achieve small power consumption; however, a large driving voltage up to 60 V is necessary to move the MEMS actuator, which makes CMOS compatible integration difficult.

Si-based carrier modulation structures such as *pin* or p-n junction are commonly researched thanks to their high CMOS compatibility, although their electro-optic effect is relatively small compared to other materials [42]. Although the optical switches using the carrier injection with a *pin* junction [93,94] is the main principle to achieve large phase modulation, its speed and power consumption suffer owing to the minority carrier response in Si compared to the modulators mainly using a *pn* junction [42]. The feasibility of the optical switches using a p-n junction is reported to achieve high-speed operation and low power consumption [95]. However, due to large carrier-induced loss for phase modulation, the crosstalk of Si *pn* and *pin* structures is worse compared to other structures [45,46,47,48].

To achieve the large phase shift using the semiconductor, III-V compound semiconductors, especially InP-based materials, have been investigated. Bulky III-V devices using a semiconductor optical amplifier (SOA) [96,97,98] or *pin* junction are demonstrated [99]. These structures can achieve relatively small crosstalk compared to Si devices thanks to the large electro-optic effect of III-V materials. However, Bulky III-V photonics has its problems, such as poor optical confinement, deep trench, and scalability [100]. To overcome these problems, a III-V on insulator structure is suggested [100]. The *pin* optical switch using this III-V on the insulator platform is reported [101]. The hybrid integration of Si and III-V is also investigated to overcome the disadvantages of III-V devices by many groups [102]. Many structures have been demonstrated such as a III-V/Si hybrid SOAs [103,104], and a III-V/Si hybrid semiconductor-insulator-semiconductor (SIS) capacitor [105]. The III-V/Si hybrid SIS structure can achieve relatively small crosstalk and small power consumption owing to its large electron phase modulation efficiency and the small leakage current of an SIS structure [105].

Recently, programmable optical switches have been actively investigated for a neuromorphic photonics application [106]. There are many reports on optical phase shifter arrays for this application, controlled by external signal [106]. For further development, to achieve efficient and multi-functional photonics switches for these applications, the non-volatile or bi-stable operation of the optical switches has been widely investigated using a phase-change material (PCM) [107], a ferroelectric BaTiO_3_ (BTO) [108], or an MEMS switch [109]. However, the high optical loss due to the metal phase of PCM and the process complexity of non-CMOS-compatible materials are the main problems with these approaches. Recently, CMOS-compatible ferroelectric materials, such as HfZrO_2_, are widely investigated for electrical devices. These CMOS compatible ferroelectric materials can also achieve efficient phase shift thanks to their large carrier accumulation enhancement using the negative capacitance effect [110]. The feasibilities of non-volatile operation for the optical switch application are also reported [111].

## 3. Discussion

In this section, we discuss the performance of modulators and switches mentioned above.

### 3.1. Optical Modulators

We summarize the performance of published phase-shifter-based optical modulators and compare them in Table 1. The material, a key for heterogeneous integration except for Si, is included as the first column name. We also include the matrix of which the performance result is based on simulation (Sim.), demonstration (Demo.), and optimization (Opt.). Performance metrics include modulation efficiency, represented by *V_π_L*, optical phase shifter loss per unit length, α, in dB/mm, the intrinsic bandwidth of the phase shifter as limited by the RC time constant or reported 3dB bandwidth of electro-optical (EO) S21 response, *f*_mod_, as well as the product of modulation efficiency and optical phase shifter loss, α*V_π_L*.

*αV_π_L* represents the DC performance of optical phase shifters. The typical Si lateral *pn* junction modulators are in the range of 15 to 20 VdB. This can be reduced to ~10 and 5 VdB by using L- or U-shaped junction while sacrificing *f*_mod_. The hybrid approaches using SiGe have relatively higher *αV_π_L*, compared to Si devices, due to high optical loss. Particularly for the SIS type, poly-Si would be the main root, which needs to be optimized considering the tradeoff between optical loss and resistance for modulation speed. The other approaches, using InGaAsP, GaAs, LN, and BTO (except plasmonic-based modulators [77]), present low *αV_π_L*. In particular, the InGaAsP SIS type and BTO/Si approaches present less than 1 VdB.

**Figure 8:**
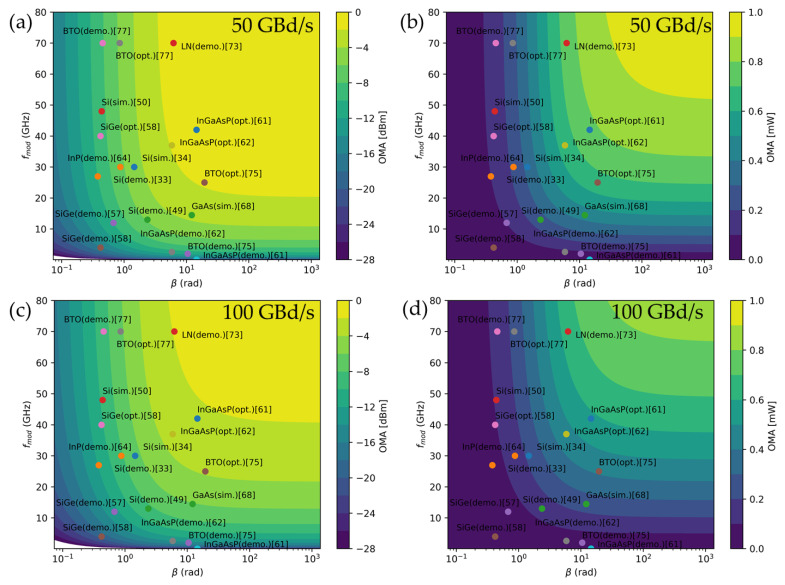
(**a**) Performance comparison of dynamic OMA for variable hybrid and Si optical phase shifters at the data rate of 50 GBd/s in (**a**) mW and (**b**) dBm scale, and 100 GBd/s in (**c**) mW and (**d**) dBm scale.

The dynamic OMA is the most important parameter for high-speed modulators in integrated photonics circuits in which the performance metrics of *V_π_L*, *α*, and *f*_mod_ are involved. We employ the simple model reported in [112] to estimate the dynamic OMA. From the reference, the maximum dynamic OMA can be written as:

(5) $$OMA_{dyn,\;max}=\left|\sin\left(\mathrm{atan}\left(\beta \right)\right)\right|\frac{e^{-\frac{1}{\beta }\mathrm{atan}\left(\beta \right)}}{\sqrt{1+r^{2}}}$$

with *β* = Δ*φ*/α where a phase shift efficiency per unit of length (rad/mm), Δ*φ,* can be obtained from *V_π_L,* and r = *f*_0_/*f*_mod_, where *f*_mod_ is intrinsic phase shifter cutoff frequency, 1/(2π*RC*), with *R* and *C*, the total resistance and capacitance of the phase shifter or cutoff frequency of entire modulator, which can be taken as a lower bound for the intrinsic phase shifter bandwidth, and *f*_0_ is half of the target baud-rate. Figure 8 shows a performance comparison of dynamic OMA for variable hybrid and Si optical phase shifters at the data rate of 50 GBd/s and 100 GBd/s in mW and dBm scale. Simply, *β*, *x*-axis, is related to *V_π_L*, meaning the higher *β* is the lower *V_π_L*, and *f*_mod_, *y*-axis, is related to dynamic performance. The maximized dynamic OMA (*OMA_dyn, max_*) calculated by Equation (5) and plotted with both parameters is shown by the contour map, as shown in Figure 8.

As expected from Table 1 and discussed, the hybrid modulators with α*V_π_L*, InGaAsP, GaAs, LN, and BTO are positioned at the high *OMA_dyn, max_* side, upper, and right-hand side in Figure 8. The hybrid photonic InGaAsP, GaAs, LN, and BTO modulators need to be designed for higher bandwidth to achieve larger *OMA_dyn, max_*. In the case of the plasmonic BTO modulator, it has fast enough *f*_mod_ while *β* is too low (particularly, plasmonic waveguide loss is too high) to be competitive with other hybrid photonic modulators. So far, the demonstrated LN modulator presents the highest OMA than other hybrid approaches even including optimized cases (e.g., optimized InGaAsP [61] or BTO [75]). The optimized InGaAsP hybrid approach presents slightly higher OMA than the LN hybrid one for 50 GBd/s, but it would be difficult to be competitive with the LN hybrid modulators for 100 Gbd/s and beyond. It would be interesting to see the RF-electrode design optimized hybrid photonic BTO modulators which are expected to achieve higher performance than hybrid LN modulators due to the strong Pockels effect.

### 3.2. Optical Switches

**Figure 9:**
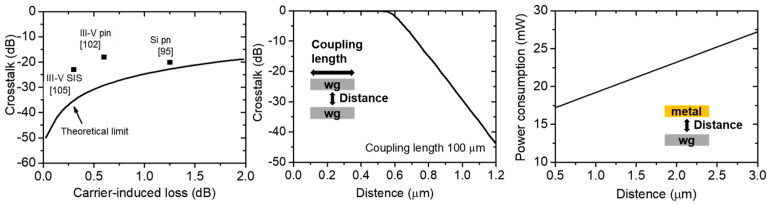
Main trade-off relationships of an optical switch using (**a**) carrier plasma dispersion effect, (**b**) MEMS actuator, and (**c**) thermo-optic effect.

As discussed in the previous section, many structures have been suggested for the large-scale optical switch array. In the case of optical switches, three different operation principles have their advantages and drawbacks. Table 2 shows the comparison of various integrated optical switches and their characteristics. Each principle has its advantages and drawbacks, which cause its trade-off relationship. In this section, we will discuss the main achievements of these devices and their characteristics. Figure 9 also show the main trade-off relationships of these structures.

The carrier-induced phase modulation has optical loss caused by the damping of the oscillation amplitude due to the energy loss of free-carrier perturbation [42,113]. This optical loss causes the crosstalk to suffer as shown in Figure 9a. Carrier-induced losses of each structure are calculated from their structural parameters and the Drude model. This trade-off relationship is determined by material properties; thus, material engineering is important to overcome this trade-off relationship. III-V compound semiconductors are a comparable candidate to achieve large phase modulation with a small optical loss [61,62,63,64,67]. The hybrid SIS structure with n-type III-V and p-type Si is especially efficient in reducing hole-induced loss of III-V materials; thus, relatively low crosstalk can be achieved [105]. Other electro-optic effects, such as QCSE [42] or the perturbation of ferroelectric materials [74], are also common alternative candidates of the plasma dispersion effect; however, there are many issues with the integration into a CMOS compatible platform.

MEMS optical switches are one of the most suitable candidates for large-scale and low-crosstalk optical switch arrays [44,46]. However, they have a structural trade-off relationship between the crosstalk and the distance of the actuator as shown in Figure 9b [92,93]. This distance also determines the driving voltage of the MEMS actuator; thus, MEMS optical switches also have a trade-off relationship between the crosstalk and the driving voltage. This trade-off relationship is one of the main issues for further CMOS-compatible integration with low supply voltage. Another main issue is its process complexity which causes high cost [78].

Thermo-optic switches also have a structural trade-off relationship between the power consumption for phase shift and the distance of the metal heater as shown in Figure 9c [114]. Thin cladding layer thickness between the waveguide and the metal heater increases the phase modulation efficiency; however, it also causes high insertion loss caused by metal [90,114].

The power penalty is one of the most powerful benchmarks for optical switches. However, the power penalty is also affected by the switch architecture; thus, we discussed the relationship between the on-state crosstalk and the optical loss induced by the structure of optical switches. The crosstalk of the thermo-optic switches and the MEMS switches is affected by the loss of waveguide, determined by the distance of a metal heater or a MEMS actuator; thus, a relatively small power penalty is achievable compared to the electro-optic effect (except using the SOA structure) [78]. Therefore, they are preferable for large-scale optical switch arrays for relatively long-haul applications. The electro-optic effect has the carrier-induced loss; thus, on-state crosstalk becomes degraded. However, as discussed in the previous section, the power consumption (in the case of MEMS switch, operation voltage) and switching speed, some of the electro-optic effects can be greatly improved compared to other structures; thus, the electro-optic effect is more suitable for further CMOS-compatible intra-chip interconnection. Therefore, each structure of the optical switches can be selectively used for its application.

## 4. Conclusions

In this paper, we reviewed heterogeneously integrated optical phase shifters composing high-speed optical modulators and low-power optical switches for the next-generation silicon photonic platform. For hybrid optical modulators, we introduced SiGe and InGaAsP based on the plasma dispersion effect, and LN and BTO based on the Pockels effect, to improve modulator performance as a hybrid material with Si. III-V materials are preferable to SiGe due to a larger change in refractive index and lower optical loss. InGaAsP can be competitive to the demonstrated LN modulator if it is optimized at the data rate of 50 GBd/s. For 100 GBd/s or beyond, the hybrid approach using LN is the most promising based on experimental results to the best of our knowledge. However, we expect that the optimized BTO with an RF electrode could be superior to the LN modulators due to its strong Pockels effect. For switches, each structure can be selectively used for its application. The thermo-optic switches and the MEMS switches are more ideal for a large-scale optical switch array with low crosstalk, which is important to achieve relatively long-haul applications, such as datacenter and inter-chip interconnection. The optical switches using the electro-optic effect are more suitable for further low-power, high-speed, and CMOS-compatible intra-chip interconnection.

## Figures and Tables

**Table 1:** Performance comparison of optical phase shifters for modulators based on loss, *V_π_L*, the product of them, and 3dB bandwidth of intrinsic RC delay or electrooptical S21 response.

Ref.	Material/Structure	Sim./Demo./Opt. ^a^	α (dB/mm)		*V*_π_*L* (Vcm)		*αV_π_L* (VdB)	*f*_mod_ (GHz)	
[34]	Si/*pn*	sim.	1.05	(at 0 V)	0.89	(0 to −2 V)	9.35	30.00	(at 0 V, Intrinsic)
[33]	Si/*pn*	demo.	1.20	(N/A)	3.00	(0 to −4 V)	36.00	36.0	(at 0 V, Intrinsic)
[49]	Si/*pn*	demo.	1.25	(at 0 V)	0.46	(at −0.5 V)	5.75	13.00	(at −2 V, EO S21)
[50]	Si/SIS	sim.	4.20	(at −1 V)	0.74	(0 to −2 V)	31.08	48.00	(at −1 V, Intrinsic)
[57]	SiGe/*pn*	demo.	3.00	(IL = 1.5 dB, de Vice 0.5 mm)	0.67	(at −0.5 V)	20.10	12.00	(at −1 V, EO S21)
[58]	SiGe/SIS	demo.	3.00	(at 1.8 V)	1.08	(0.9 V to 1.8 V)	32.40	4.00	(at 0.9 V, EO S21)
[58]	SiGe/SIS	opt.	3.00	(at 1.8 V)	1.08	(0.9 V to 1.8 V)	32.40	40.00	(expected by sim., EO S21)
[62]	InGaAsP/ SIS	demo.	2.60	(at −1.5 V)	0.09	(in accumulation mode)	2.34	2.60	(at 1.5 V, EO S21)
[62]	InGaAsP/ SIS	opt.	2.60	(at −1.5 V)	0.09	(in accumulation mode)	2.34	37.00	(at 1.5 V, intrinsic)
[61]	InGaAsP/ SIS	demo.	2.00	(at 0 V)	0.05		0.94	0.10	(EO S21)
[61]	InGaAsP/ SIS	opt.	2.00	(at 0 V)	0.05		0.94	42.00	(Intrinsic)
[64]	InP/SIS	demo.	1.20	(at 0 V)	1.30	(0 to 4 V)	15.60	30.00	(at 0 V, 250 um, EO S21)
[67]	GaAs/*pn*	sim.	1.60	(at 0 V)	0.07	(0 to −0.3 V)	1.12	14.47	(0 V, Intrinsic)
[73]	LN	demo.	0.10		2.20		2.20	70.00	(EO S21)
[75]	BTO	demo.	0.57		0.23		1.30	2.00	(EO S21)
[75]	BTO	opt.	0.30		0.23		0.70	25.00	(EO S21, measured value using micro-ring modulator)
[77]	BTO	demo.	1500.00		0.002		30.00	70.00	(EO S21)
[77]	BTO	opt.	800.00		0.002		16.00	70.00	(EO S21)

^a^ Simulation (Sim.), Demonstration (Demo.), and Optimization (Opt.).

**Table 2:** Comparison of various integrated optical switches and their characteristics.

Ref	Switching Principle	Structure	Reported Largest Scalability	Speed	Crosstalk (dB)	Insertion Loss (dB)	Power Consumption
[82,83,84,85]	Thermo-optic	Metal heater	32 × 32	~30 μs	>35	8.4	1.9 W
[86,87,88,89]	MEMS	MEMS actuator	240 × 240	Sub-μs	>60	<6.5	(20–40 V) ^a^
[83,94,95]	Electro-optic	Si *pin*	32 × 32	<10 ns	>10	<5	1.2 W
[95]		Si *pn*	2 × 2	Sub-ns	>10	<11.5	4.7 mW
[97,98,99]		III-V SOA	16 × 16	<3.3 ns	>51.6	-	1.26 W
[100,102]		III-V *pin*	8 × 8	<5 ns	>18	<6.3 (sim.)	-
[105]		III-V/Si hybrid SIS	2 × 2	<20 ns	>23	>0.07 (sim.)	0.18 W

^a^ Relatively high voltage is necessary for operating.
